# A self-attention based message passing neural network for predicting molecular lipophilicity and aqueous solubility

**DOI:** 10.1186/s13321-020-0414-z

**Published:** 2020-02-21

**Authors:** Bowen Tang, Skyler T. Kramer, Meijuan Fang, Yingkun Qiu, Zhen Wu, Dong Xu

**Affiliations:** 1 Fujian Provincial Key Laboratory of Innovative Drug Target Research, School of Pharmaceutical Sciences, Xiamen University, Xiamen, 361000 China; 2 Department of Electrical Engineering and Computer Science, Informatics Institute, and Christopher S. Bond Life Sciences Center, University of Missouri, Columbia, MO 65211 USA

**Keywords:** Message passing network, Attention mechanism, Deep learning, Lipophilicity, Aqueous solubility

## Abstract

Efficient and accurate prediction of molecular properties, such as lipophilicity and solubility, is highly desirable for rational compound design in chemical and pharmaceutical industries. To this end, we build and apply a graph-neural-network framework called self-attention-based message-passing neural network (SAMPN) to study the relationship between chemical properties and structures in an interpretable way. The main advantages of SAMPN are that it directly uses chemical graphs and breaks the black-box mold of many machine/deep learning methods. Specifically, its attention mechanism indicates the degree to which each atom of the molecule contributes to the property of interest, and these results are easily visualized. Further, SAMPN outperforms random forests and the deep learning framework MPN from Deepchem. In addition, another formulation of SAMPN (Multi-SAMPN) can simultaneously predict multiple chemical properties with higher accuracy and efficiency than other models that predict one specific chemical property. Moreover, SAMPN can generate chemically visible and interpretable results, which can help researchers discover new pharmaceuticals and materials. The source code of the SAMPN prediction pipeline is freely available at Github (https://github.com/tbwxmu/SAMPN).

## Introduction

Accurate and reliable prediction of molecular properties is an important ingredient in drug discovery and chemical material projects [1–3]. Characterizing quantitative structure-bioactivity/structure–property relationships (QSAR/QSPR) of compounds has always been a hot topic in medicinal and material chemistry [2, 4], but such relationships are usually difficult to elucidate with heuristic rules or empirical measurements. Machine learning (ML) methods, such as random forests (RF) and support vector machines (SVM), have aided the discovery process of new chemical drugs and materials [2, 5, 6]. For example, random forests models with atom pair descriptors have been used by many pharmaceutical companies to construct QSAR models [7], and Bayesian optimization models have been used to design nanostructures for phonon transport [8]. More recently, however, neural-network-based methods have greatly accelerated this field and will be briefly discussed below.

Many ML methods first convert chemical molecules into a computer-interpretable representation, utilizing physicochemical properties from experimental/computational measurements [9] or by using molecular fingerprints. Physiochemical properties include mass, charge, refractivity, and many other physical features of the molecules. The most widely used molecular conversion, however, is the molecular fingerprint, which encodes a molecular structure into a series of binary digits (a bit vector) [10] based on substructures that may or may not be pre-defined, depending on the class of fingerprints being used. For example, extended-connectivity fingerprints (ECFP) can split one molecule into many substructures (not pre-defined) and encode all of them into just one bit vector with different identifiers [11]. ENREF_10 Alternatively, bit vectors may be extended into count vectors that indicate the number of each substructure found in the molecule, not just its presence/absence.

Compared to the previously-mentioned traditional methods, artificial neural networks (ANNs) have become increasingly popular in predicting molecular properties. For example, a three-layered ANN with E-state indices was used to predict aqueous solubility of organic molecules [15]. More recently, graph-based networks were applied to predict lipophilicity and solubility [16]. These network-based models have shown impressive results and made good contributions for developing new methods.

**Fig. 1:**
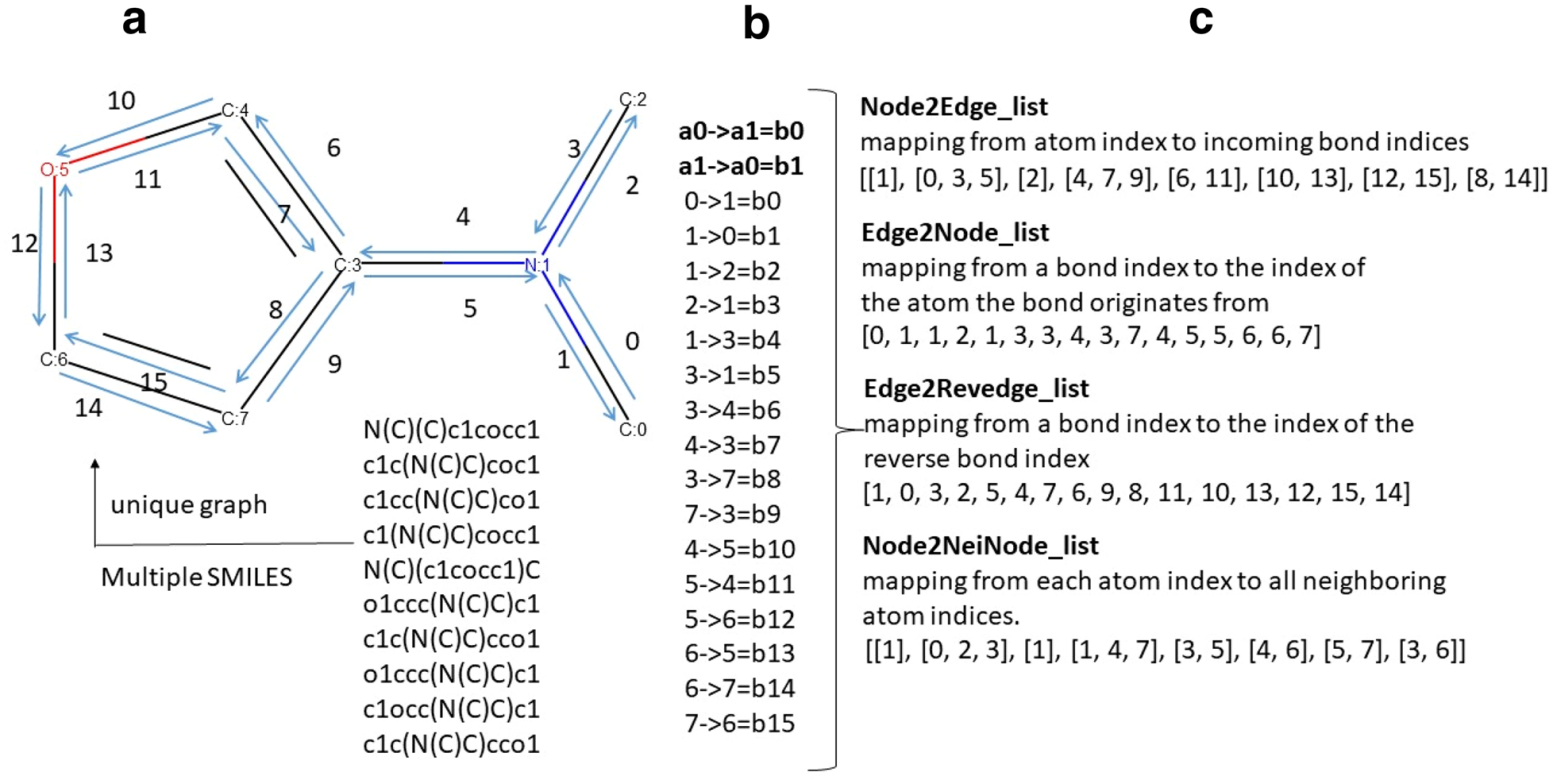
Conversion of a chemical structure into a mathematical graph. **a** A chemical structure usually has a unique graph but multiple SMILES strings. **b** Relationship list between node indices and edge indices, which are converted from the chemical graph. **c** The lists of Node2Edge, Edge2Node, Edge2Revedge and Node2NeiNode, derived from (**b**)

Fixed fingerprint feature extraction rules of molecules are useful to accurately reflect underlying chemical substructures, though these may not be the best-suited representation for all tasks. Hence, researchers have to spend much time and effort to carefully determine which features are most relevant to their models. This is especially problematic with the utilization of physical features, which may require advanced variable selection techniques or a high-level of empirical knowledge. In contrast, some deep learning networks based on the simplified molecular input line entry system (SMILES) [12] codes can automatically learn the molecular features [13, 14]. However, this may cause the model to focus on the SMILES grammar and not the implicated molecular structure. This limitation of the SMILES-based deep learning models is hard to avoid as the SMILES representation is not designed to capture molecular similarity. Generally, molecules with similar chemical structures can be encoded into very different SMILES strings. Even for the same molecular structure, there are often non-unique SMILES strings as Fig. 1A displays. Though the process of generating canonical SMILES is well known, the process is inconsistent among chemical toolkits. For example, the ‘canonical’ SMILES code for caffeine is CN1C=NC2=C1C(=O)N(C)C(=O)N2C according to RDKit, Cn1cnc2c1c(=O)n(C)c(=O)n2C according to Obabel, and CN1C=NC2=C1C(=O)N(C(=O)N2C)C according to PubChem.

Using the natural chemical graph instead of the SMILES representation may be more suitable for chemical property predictions. Briefly, a graph consists of nodes and edges that connect two or more nodes to one another. Analogously, a chemical graph considers atoms as nodes and bonds as the edges connecting atoms to one another. Our formulation considers these edges as bidirectional, meaning that the bond connecting atom A to atom B is the same as the bond connecting atom B to atom A. An example chemical graph can be seen in Fig. 1a.

Essential chemical properties such as molecular validity are more easily represented in two-dimensional chemical graphs than linear SMILES. Unlike SMILES codes chemical graphs are invariant to molecule permutations, i.e., one molecular structure has one graph but multiple SMILES representations. Recently, graph-based deep learning models are reported in QSAR and QSPR studies [7, 17–21]. However, according to these references, predictions are difficult to interpret, since most neural networks act as black boxes [22].

In this paper, we describe a self-attention-based message-passing neural network (SAMPN) model, which is a modification of Deepchem’s MPN [16] and is state-of-the-art in deep learning. It directly learns the most relevant features of each QSAR/QSAPR task in the learning process and assigns the degree of importance for substructures to improve the interpretability of prediction. Our SAMPN graph network utilizes the chemical graph structure described above, where each edge is derived from the chemical bond and each atom is the node. Both our message passing neural network (MPN) and SAMPN model can be used as multi-target models (Multi-MPN or Multi-SAMPN), which can learn not only the relationship between chemical structures and properties, but also the relationship between intrinsic attributes of molecules. To demonstrate our computational methods, we chose lipophilicity and aqueous solubility as the target properties as they were very important chemical descriptors that pervade every aspect of bioactivity, drug metabolism and pharmacokinetic (DMPK) profiles [23].

To our knowledge, this is the first time that a model like SAMPN has been used to predict chemical properties from experimental data for QSPR studies. The results from our experiments demonstrate that our SAMPN network yields superior performance relative to traditional ML-based models and previous deep-learning models (i.e., Deepchem’s MPN [16]). Furthermore, the predictions of SAMPN are easily understood and visualized, since the integrated attention mechanism can color the atoms of the molecule based on their contributions to the property of interest.

## Methods and materials

### Datasets and data process

Datasets of molecular lipophilicity and aqueous solubility were used for developing and testing our method. Lipophilicity is usually quantified by the *n*-octanol/water partition coefficient P and preferentially displayed in a logarithmic form as logP. The raw lipophilicity data was downloaded from CHEMBL3301361 deposited by AstraZeneca [24] and includes 4200 molecules. Aqueous solubility is the saturated concentration of the chemical in the aqueous phase, which is usually displayed with unit log(mol/L) and is represented as logS. This dataset was downloaded from the online chemical database and modeling environment (OCHEM) [25] and includes 1311 experimental records. The dataset distributions are plotted in Additional file 1: Fig. S1.

As both datasets are small relative to the typical size requirements of deep learning models, we use tenfold stratified cross-validation [13, 23, 35], where each dataset was randomly split into a training and validation set (80% and 10%, respectively) for parameter selection and a test dataset (10%) for model comparisons. Then, we repeated all experiments three times with different random seeds. This process ensures that the model does not simply memorize the training and is capable of generalizing to new molecules.

For the initial data preprocessing, duplicate molecules were removed so that each chemical structure in the data was unique, while the maximum one of the related properties was kept. Molecules unrecognized by RDkit (version 2019.3) [26], a cheminformatics toolkit implemented in Python, were also deleted. Only two columns (‘smiles’ and ‘experimental value’) were kept as the input data to our models. Each downloaded SMILES representation was then converted into a directed graph before training the SAMPN model using the MPN encoder, which was adapted from Deepchem and Chemprop [27, 28]. The directed graphs were mainly composed of index lists of nodes and edges shown in Fig. 1c. Take the substructure of N–C as an example: a chemical bond between the N and C atoms can derive two edges (C:0 → N:1 and N:0 → C:1). The number of nodes is equal to the number of atoms and the number of edges is always double the number of bonds, since we consider edges to be bidirectional.

### Message passing network encoder

**Fig. 2:**
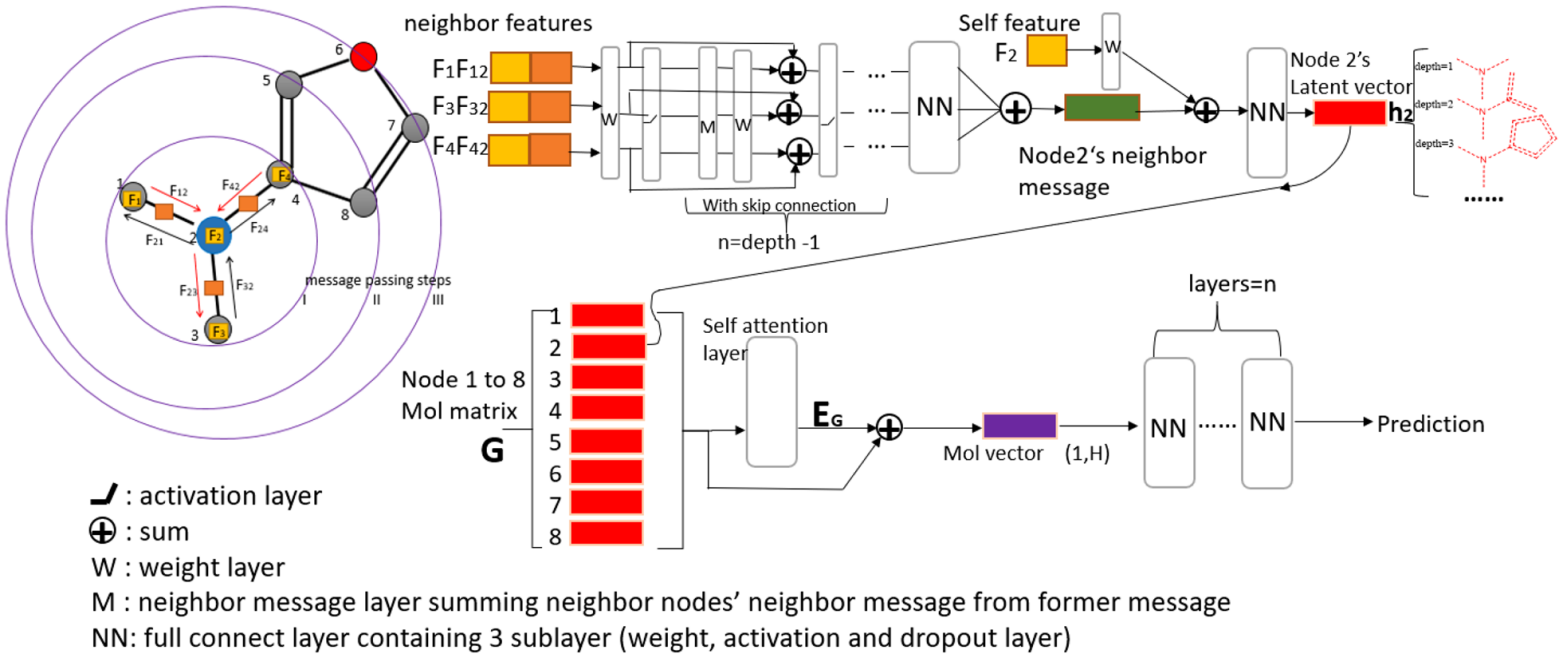
Representation of SAMPN architecture. The main part of the MPN encoder converts the neighbor features to a molecule matrix, then followed by a self-attention layer and fully connected networks to make a final prediction

Instead of manually selected features, using molecular graph structures directly was first reported in 1994 [29]. In recent years, graph-based methods have been used to analyze various aspects of chemical systems [14, 30] and compare with fingerprints [31]. Graph-based models provide a natural way to describe chemical molecules, where atoms in the molecule are equivalent to nodes and chemical bonds to the edges in a graph. The message-passing network is a variant of the graph-theoretical approaches, which gradually merges information from distant atoms by extending radially through bonds as displayed in Fig. 2. Those passing messages were used to encode all substructures of a molecule by an adaptive learning approach, which extracts useful representations of molecules suited to the target predictions.

The message passing network encoder works as follows in Eqs. (1–3). The passing message *M* from atom *x* to atom *y* in the *d*-th iteration (message passing depth) is calculated as follows:

1 $$M_{xy}^{d = 1} = Re\left( {W_{inp} \cdot f_{x} f_{y} } \right)$$

2 $$M_{xy}^{d > 1} = Re\left( {W_{inp} \cdot f_{x} f_{y} + W_{h} \sum\limits_{z \in N\left( x \right)\backslash y} {M_{zx}^{d - 1} } } \right)$$

Here, *Re* is the activation function (Relu). $W_{inp}$ and $W_{h}$ are the learned weight matrices. As we use the edge-dependent neural network to pass a message, node feature *f*_*x*_ is concatenated with edge feature *f*_*xy*_ to form the merged node-edge feature *f*_*x*_*f*_*xy*_. Node feature *f*_*x*_, is derived by atom type, formal charge, valence, and aromaticity. Similarly, edge feature *f*_*xy*_ is derived from bond order, ring status and direction connection. The definitions of node *f*_*x*_ features and edge *f*_*xy*_ features are displayed in Table 1. The initial message $M_{xy}^{d = 1}$, which *x* sends to *y*, is generated from the merged node-edge feature *f*_*x*_*f*_*xy*_ by a neural network as described in Eq. (1).

**Table 1:** Descriptions of node and edge features

Attribute	Description	Dimension
Node		
Atom type	All currently known chemical elements	118
Degree	Number of heavy atom neighbors	6
Formal charge	Charge assigned to an atom (− 2, − 1, 0, 1, 2)	5
Chirality label	R, S, unspecified and unrecognized type of chirality	4
Hybridization	sp, sp^2^, sp^3^, sp^3^d, or sp^3^d^2^	5
Aromaticity	Aromatic atom or not	1
Edge		
Bond type	Single, double, triple, or aromatic	4
Ring	Whether the bond is in a ring	1
Bond stereo	Nature of the bond’s stereochemistry (none, any, Z, E, cis, or trans)	6

In a chemical graph, atoms denote the node set *x∈V,* and bonds denote the edge set *(x,y)∈E.* Each edge has its own direction in the SAMPN model. *N(x)* or *N(y)* stands for the group of neighbor nodes of *x* or *y*, respectively. $z \in N\left( x \right)\backslash y$ means the neighbors of *x* do not contain *y*. Node *x* is allowed to send a message to a neighbor node *y* only after node *x* has received messages from all neighbor nodes except *y*. We use the skip connection in the message passing steps as in Fig. 2 (displayed in between neighbor features and self-features). This skip connection allows the message to pass a very long distance without vanishing gradient problem when using backpropagation. The generated messages exchange and update based on the merged node-edge feature and the previous message passing step as Eq. (2) defined.

The latent vector $h_{y}$ of each node, take Node 2’s latent vector $h_{2}$ as an example in Fig. 2, is obtained by aggregating its neighbor messages in Eq. (3) after the message-passing process:

3 $$h_{y} = Re\left( {W_{o} \left( {W_{ah} \cdot f_{y} + \sum\limits_{z \in N\left( y \right)} {M_{zy}^{d} } } \right)} \right)$$

where, $h_{y}$ captures the local chemical structure features based on the passing depth, and $W_{o}$ and $W_{ah}$ are the learned weight matrices. More detailed information of SAMPN algorithm can be found in Additional file 1: Table S1 in Supporting Materials. Applying the above Eqs. (1–3) on a chemical graph generates the final graph representation *G* = {*h*_*1*_ … *h*_*i*_ … *h*_*n*_}, which combines with the self-attention mechanism and fully-connected neural networks to make the final prediction.

### Self-attention mechanism

All hidden states of a node are directly combined into a single vector, which may not make the difference among the learned features explainable [32]. A better way is to apply the attention mechanism to obtain a context vector for the target node by focusing on its neighbors and local environment. Take Node 2 as an example (the blue node in Fig. 2), after several message passing steps, Node 2 has hidden state h_2_, which represents the substructure centered at Atom 2. Meanwhile, all the rest nodes have the same process and h_n_ represents the substructure centered at Atom n. Since different substructures have different contribution to the molecular property, we can use the attention mechanism to capture the different influences of substructures in contributing to the target molecular property.

A self-attention layer is then added to identify the relationship between the substructure contribution to the target property of a molecule. A dot-product attention algorithm was implemented to take the whole molecular graph representation G as the input. The self-attentive weighted molecule graph embedding can be formed as follows:

4 $$W_{att} = softmax\left( {G \cdot G^{T} } \right)$$

5 $$E_{G} = W_{att} \cdot G$$

where *W*_*att*_ is the self-attention score that implicitly indicates the contribution of local chemical graph to the target property. As *G* = {*h*_*1*_ … *h*_*i*_ … *h*_*n*_}, each row of *W*_*att*_ is the attention weight between the *i*-th atom and the rest atoms in the molecule. *E*_*G*_ is the attentive embedding matrix, where each row corresponds to the attention weighted hidden vector of the node. Then, the global average pooling is used on the sum of *G* and *E*_*G*_ to get the molecule latent vector as Fig. 2 shows in the purple rectangle. Finally, the latent vector is combined with several layers of fully connected networks for the target property prediction.

### Model training and hyperparameter optimization

The code for the MPN encoder was mainly adapted from Deepchem and Chemprop [27, 28]. Both the MPN encoder and self-attention mechanism were implemented with Python and Pytorch version 1.0, an open-source framework for deep learning [33]. MPN, Multi-MPN, SAMPN and Multi-SAMPN models were trained with the Adam optimizer using the same learning rate schedule in [34].

Multiple metrics were used to evaluate the performance of our models: mean absolute error (MAE), root mean squared error (RMSE), mean squared error (MSE), coefficient of determination (R^2^) and Pearson correlation coefficient (PC). Lower values of MAE, MSE, and RMSE indicate better predictive performance. Conversely, higher values for PC and R^2^ indicate better models or better fits for the data. While some of these metrics tell the same story, the inclusion of all of these values may provide a rich benchmark for future studies.

A grid search algorithm was used to adjust the hyperparameters with Hyperopt package version 0.1.2 [35]. Table 2 shows the hyperparameters to be optimized and the search space. We chose RMSE on the validation set as the metric to find the most suitable combination of the hyperparameters within the search space. In the lipophilicity-QSPR task, one of the best combinations of hyperparameters was {‘activation’: ‘ReLU’; ‘depth’: 4; ‘dropout’: 0.25; ‘layers of fully connected networks’: 2; ‘hidden size’: 384}. All the message passing neural network models (MPN, SAMPN, Multi-MPN and Multi-SAMPN) utilized the above hyperparameters to test the final performance with using the tenfold stratified cross-validation on the whole dataset.

**Table 2:** Hyperparameters optimization for MPN and SAMPN

MPN and SAMPN	Hyperparameters	Range (interval)
	Activation function	Tanh, ELU, LeakyReLU ReLU, PReLU, SELU
	Steps of message passing	2–6 (1)
	Graph embedding size	32–512 (32)
	Dropout rate	0.0–0.4 (0.05)
	Layers of fully connected network	1–3 (1)

In addition to using the published results from Deepchem’s MPN, we also built a pure MPN model to establish a baseline without the self-attention and all the rest configurations were kept the same to SAMPN. To compare the single-task and multi-target based deep learning network, we built the multi-MPN and multi-SAMPN. The multi-target-based model used a merged molecule dataset from ‘Lipophilicity’ and ‘Water Solubility’ as described in Supporting Materials. All the used parameters were kept the same between MPN and SAMPN.

### Random forest

To compare our SAMPN method with the traditional machine learning methods, we chose a random forest model as the baseline. Random forest (RF) [36] is a supervised learning algorithm with an ensemble of decision trees generated from a bootstrapped (bagged) sampling of compounds and features. It is widely used in the traditional structure–property relation research [37], and was considered as a “gold standard” according to its robustness, easy usage and high prediction accuracy in structure–property relationship research [38]. Here, the ECFP with a fixed length of 1024 [12] was used with the RF model, which was implemented in Python 3.6.3 [39] with the package Scikit-learn, version 0.21.2 [40]. For the RF model, more trees generally increase performance and make predictions more stable, but it also slows down the computation heavily. We set 500 trees for a good balance point as suggested in [16] for most QSPR studies.

## Results and discussion

### Lipophilicity and solubility prediction

**Fig. 3:**
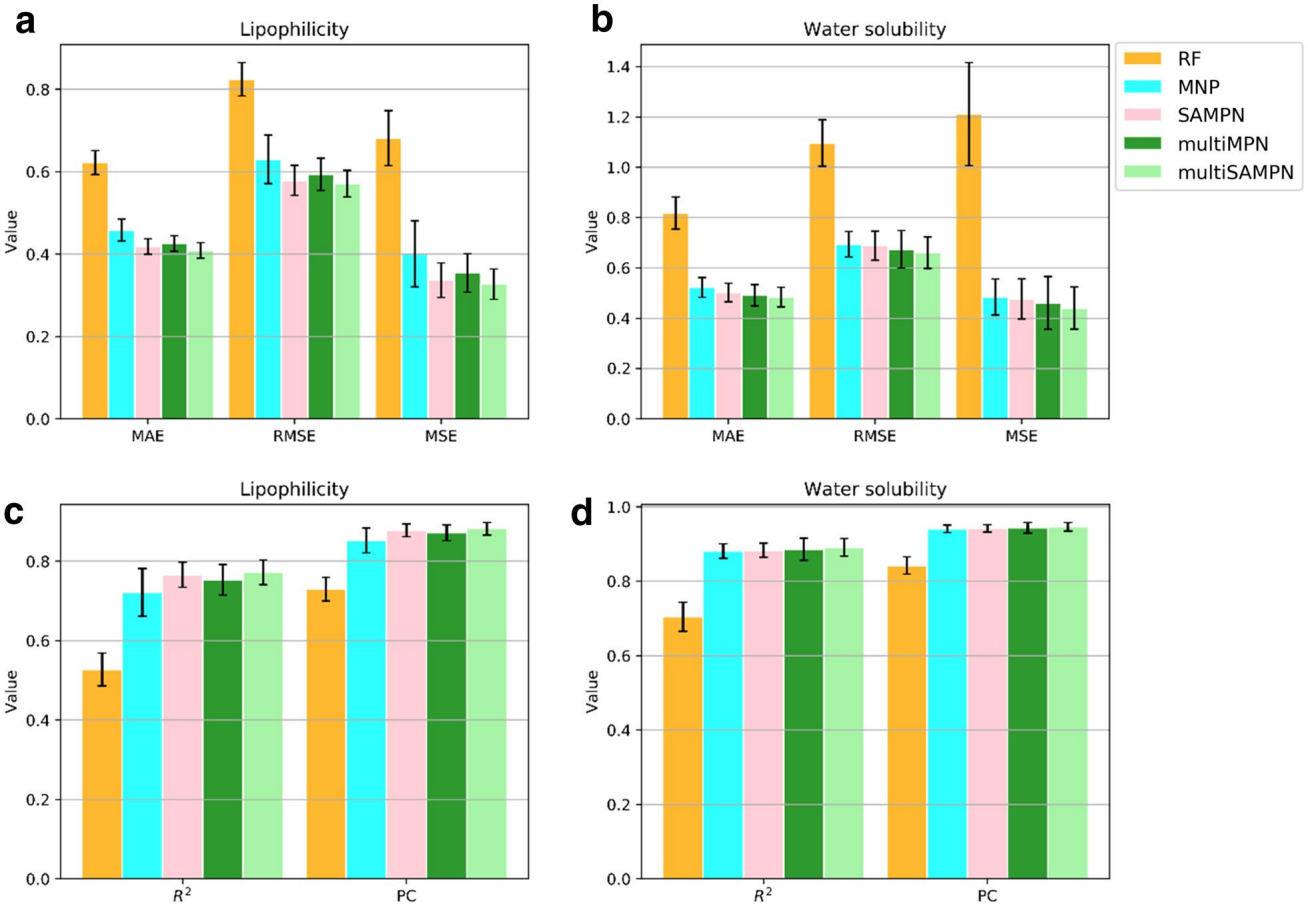
Models’ performance on lipophilicity (**a**, **c**) and aqueous solubility (**b**, **d**) with the same tenfold stratified cross-validation. Error bars represent standard deviations

In each QSPR task, we built RF, MPN, SAMPN, multi-MPN and multi-SAMPN models to explore the relationship between the target property and the molecular structure. For the lipophilicity prediction, both single-target based and multiple-target based model have good performance according to RMSE (SAMPN: 0.579 ± 0.036; Multi-SAMPN: 0.571 ± 0.032). Without the self-attention mechanism, the performance of MPN decreased as Table 3 and Fig. 3a show. Nevertheless, the result of our new formulation of the MPN or Multi-MPN was still much better than the one from the MPN version of Deepchem (0.719 ± 0.031) [16], and performance increased even more with the inclusion of the attention mechanism. Our MPN model is different from Deepchem in that we did not use any recurrent neural networks (RNN) in our network architecture, which improved the speed of our MPN model in training.

**Table 3:** Models’ performance (root-mean-square error) on lipophilicity database

Dataset (size)	Model	RMSE
Lipophilicity (4200)	RF	0.824 ± 0.041
	MPN (Deepchem)^a^	0.630 ± 0.059
	MPN (Deepchem)^b^	0.652 ± 0.061
	MPN	0.630 ± 0.059
	SAMPN	0.579 ± 0.036
	Multi-MPN	0.594 ± 0.039
	*Multi-SAMPN*	*0.571 ± 0.032*
Water solubility (1311)	RF	1.096 ± 0.092
	MPN (Deepchem-1128)^a^	0.580 ± 0.030
	MPN (Deepchem)^b^	0.676 ± 0.022
	MPN	0.694 ± 0.050
	SAMPN	0.688 ± 0.057
	Multi-MPN	0.674 ± 0.074
	*Multi-SAMPN*	*0.661 ± 0.063*

Italics represents the best performance in the results

^a^Values were reported in [16]. In the lipophilicity prediction, we use the same dataset with Deepchem. In the water solubility prediction, our used dataset is larger than Deepchem used (1128 molecules)

^b^Values were calculated from the same data and the same stratified cross-validation protocol in our work

For the solubility prediction, message passing based networks also greatly improved the performance over the traditional model (RF) as displayed in Table 3 and Fig. 3b. The MPN from Deepchem also displayed a good performance (0.580 ± 0.030 RMSD) on the water solubility prediction [16]; however, they used a small water solubility dataset (1128 molecules). For comparison purposes, we used their default setting MPN (Deepchem) on our water solubility data (1311 molecules) with our stratified cross-validation protocol. The scripts of the detail calculation process were available in our Github repository. After that, MPN (Deepchem) shows similar performance (0.676 ± 0.022) with our MPN (0.694 ± 0.050). Based on our model results (performance: SAMPN > MPN; Multi-SAMPN > Multi-MPN), the self-attention mechanism can improve the performance of message passing neural networks in both lipophilicity and solubility prediction. And multi-target models have better performance than single task-based model (performance: Multi-SAMPN > SAMPN; Multi-MPN > MPN). In our study, no matter choosing which metric as Fig. 3 displayed, we can get the same conclusion as we motioned above. The better predictive performance of the multi-target model is probably from the benefit that Multi-SAMPN or Multi-MPN can use the learned feature from lipophilicity to help the solubility prediction, and vice versa. It is worth mentioning that Multi-SAMPN or Multi-MPN can predict the lipophilicity and aqueous solubility simultaneously rather than step by step prediction like SAMPN or MPN. Although our case indicates that a multi-target model performs better than the single-target model, it requires more studies to show whether this is general, since our case only used only one lipophilicity and water solubility dataset.

### Visualize the attention

While higher prediction accuracy is always desirable, the ability to interpret a QSPR model is also important. Model comparison and interpretation can be facilitated by a visualization technique, making it possible to identify the learned features that drive compound property predictions. In the SAMPN model, we can obtain the attention weight scores from the self-attention mechanism. For a specific molecule, we obtain the difference between each atom’s weight score and the average attention weight of the molecule. We define the above difference as the attention coefficient of each atom and those attention weight coefficients are very useful to gain insight into which parts of a molecule increase the target molecular property and which decrease it.

**Fig. 4:**
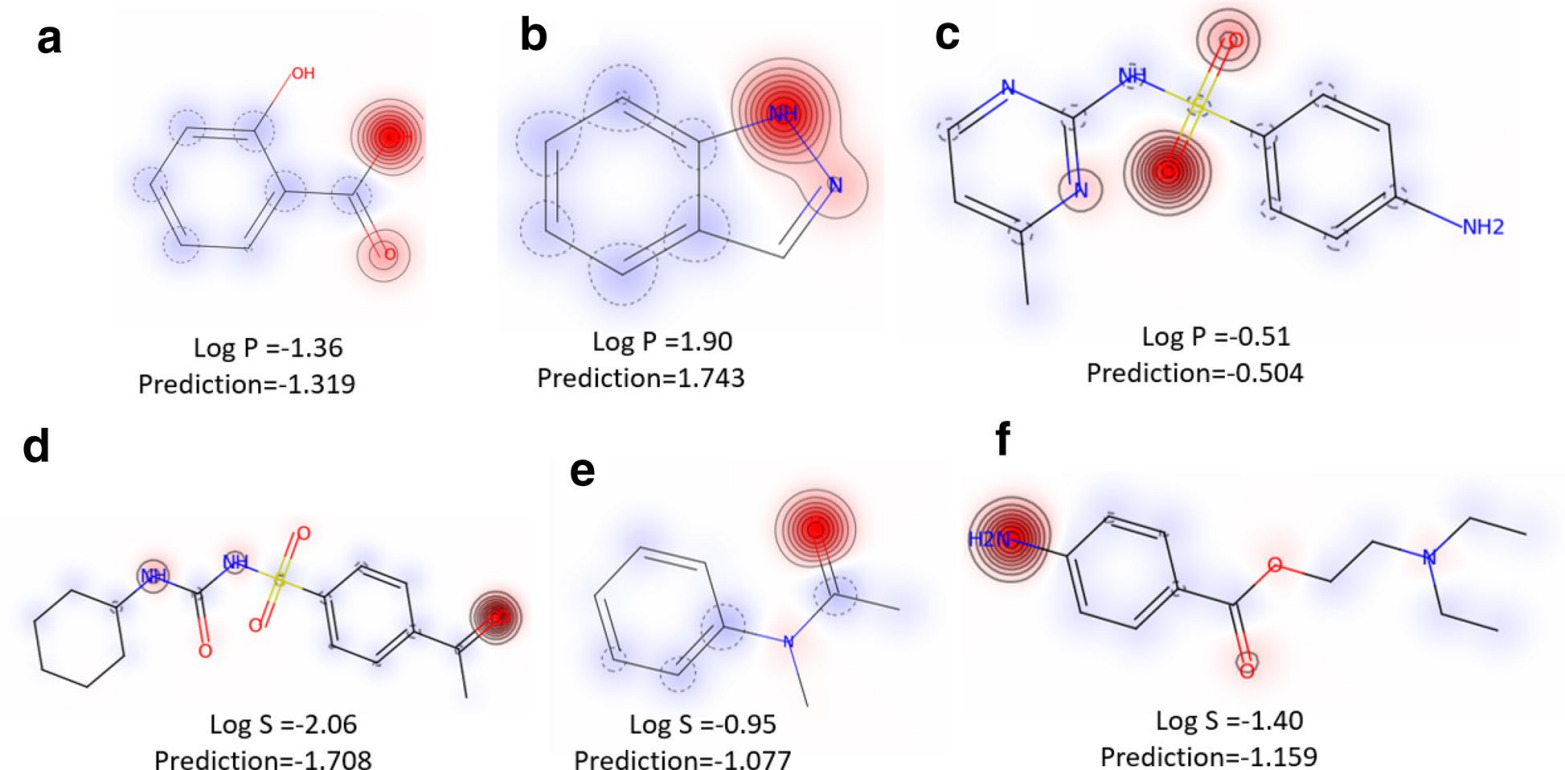
Heat map molecule coloring on lipophilicity (**a**–**c**) and solubility (**d**–**f**). **a**–**c** Red indicates a predicted anti-lipophilic feature and blue indicates a predicted lipophilic feature. **d**–**f** Red indicates a predicted soluble feature and blue indicates a predicted anti-soluble feature

By using heat map coloring on each molecule (such as in Fig. 4a–f), it is easy to see which parts of molecule play a more important role in the lipophilicity or water solubility of molecule. The lipophilicity and solubility heat maps are helpful for chemists to optimize the lipophilicity and solubility of a particular molecule. Consider Fig. 4b, a depiction of 1H-indazole after using our model. This molecule has a relatively high lipophilicity, as it has a large π-electron-conjugated system in its fused aromatic ring. However, the nitrogen-containing section of the molecule displays strong anti-lipophilic properties relative to the rest of the molecule. This may, in part, be due to nitrogen’s contribution (as ‘N’ or ‘NH’) to a hydrogen bonding-network with its surroundings. Thus, altering 1H-indazole to disrupt that potential network may increase the molecule’s lipophilicity. To test this hypothesis, we used SAMPN to predict the lipophilicity of benzo[*d*]isothiazole (Additional file 1: Fig. S2), the molecule made by exchanging the ‘NH’ of 1H-indazole with ‘S’ (sulfur). As expected, this change did increase the molecule’s lipophilicity. Another example is the primary amine group in Fig. 4f, which can easily form hydrogen bonds with water molecules. This is reflected in red for a predicted soluble feature.

## Conclusions

In this work, we have proposed a self-attention-based message passing neural network for identifying the relationship between molecular lipophilicity/solubility and structure. Our SAMPN model outperforms the conventional random forests and the previous graph neural network-based model. By applying the attention mechanism, SAMPN can provide some insights on the atomic sources of lipophilicity/solubility, which is different from black box approaches that most machine learning and deep learning methods used. The results from SAMPN are easy to understand by coloring the attention scores directly on the molecular graph, which is useful as a guide to adjust the lipophilicity or solubility of one molecule. In addition, our message-passing neural networks can be easily trained as a multi-target model, which makes it better in computational efficiency and predictive performance. With this approach and our case study, we believe our method can be applied to other quantitative structure–property relationship studies and help chemists optimize molecular properties directly from the chemical structures.

## Supplementary information

**Additional file 1.** The Supporting Information can be found in the supporting documents. The source code and the prepared datasets are available in the SAMPN Github repository (https://github.com/tbwxmu/SAMPN).

## Data Availability

All data sets and code are available at GitHub: https://github.com/tbwxmu/SAMPN.

## References

[1] Hansen K, Biegler F, Ramakrishnan R, Pronobis W, Von Lilienfeld OA, Müller K-R, Tkatchenko A (2015). Machine learning predictions of molecular properties: accurate many-body potentials and non-locality in chemical space. J Phys Chem Lett.

[2] Cherkasov A, Muratov EN, Fourches D, Varnek A, Baskin II, Cronin M, Dearden J, Gramatica P, Martin YC, Todeschini R (2014). Qsar modeling: where have you been? Where are you going to?. J Med Chem.

[3] Chen H, Engkvist O, Wang Y, Olivecrona M, Blaschke T (2018). The rise of deep learning in drug discovery. Drug Discov Today.

[4] Le T, Epa VC, Burden FR, Winkler DA (2012). Quantitative structure-property relationship modeling of diverse materials properties. Chem Rev.

[5] Gómez-Bombarelli R, Aguilera-Iparraguirre J, Hirzel TD, Duvenaud D, Maclaurin D, Blood-Forsythe MA, Chae HS, Einzinger M, Ha D-G, Wu T (2016). Design of efficient molecular organic light-emitting diodes by a high-throughput virtual screening and experimental approach. Nat Mater.

[6] Mannodi-Kanakkithodi A, Pilania G, Huan TD, Lookman T, Ramprasad R (2016). Machine learning strategy for accelerated design of polymer dielectrics. Sci Rep.

[7] Feinberg EN, Sheridan R, Joshi E, Pande VS, Cheng AC (2019) Step change improvement in Admet prediction with Potentialnet deep Featurization. arXiv preprint arXiv:190311789 10.1021/acs.jmedchem.9b02187 32286824

[8] Ju S, Shiga T, Feng L, Hou Z, Tsuda K, Shiomi J (2017). Designing nanostructures for phonon transport via bayesian optimization. Phys Rev X.

[9] Hansch C, Maloney PP, Fujita T, Muir RM (1962). Correlation of biological activity of phenoxyacetic acids with hammett substituent constants and partition coefficients. Nature.

[10] Riniker S, Landrum GA (2013). Open-source platform to benchmark fingerprints for ligand-based virtual screening. J Cheminform.

[11] Rogers D, Hahn M (2010). Extended-connectivity fingerprints. J Chem Inf Model.

[12] Weininger D (1988). Smiles, a chemical language and information system. 1. Introduction to methodology and encoding rules. J Chem Inf Comput Sci..

[13] Olivecrona M, Blaschke T, Engkvist O, Chen H (2017). Molecular de-novo design through deep reinforcement learning. Journal of cheminformatics.

[14] Li X, Yan X, Gu Q, Zhou H, Wu D, Xu J (2019). Deepchemstable: chemical stability prediction with an attention-based graph convolution network. J Chem Inf Model.

[15] Tetko IV, Tanchuk VY, Kasheva TN, Villa AE (2001). Estimation of aqueous solubility of chemical compounds using E-state indices. J Chem Inf Comput Sci.

[16] Wu Z, Ramsundar B, Feinberg EN, Gomes J, Geniesse C, Pappu AS, Leswing K, Pande V (2018). Moleculenet: a benchmark for molecular machine learning. Chem Sci.

[17] Réti T, Sharafdini R, Dregelyi-Kiss A, Haghbin H (2018). Graph irregularity indices used as molecular descriptors in qspr studies. MATCH Commun Math Comput Chem.

[18] Sarkar D, Sharma S, Mukhopadhyay S, Bothra AK (2016) Qsar Studies of Fabh inhibitors using graph theoretical & quantum chemical descriptors. Pharmacophore 7

[19] Shao Z, Hirayama Y, Yamanishi Y, Saigo H (2015). Mining discriminative patterns from graph data with multiple labels and its application to quantitative structure–activity relationship (Qsar) models. J Chem Inf Model.

[20] Wang X, Li Z, Jiang M, Wang S, Zhang S, Wei Z (2019). Molecule property prediction based on spatial graph embedding. J Chem Inf Model.

[21] Liu K, Sun X, Jia L, Ma J, Xing H, Wu J, Gao H, Sun Y, Boulnois F, Fan J (2019). Chemi-Net: a molecular graph convolutional network for accurate drug property prediction. Int J Mol Sci.

[22] Goulon A, Picot T, Duprat A, Dreyfus G (2007). Predicting activities without computing descriptors: graph machines for Qsar. SAR QSAR Environ Res.

[23] Arnott JA, Planey SL (2012). The influence of lipophilicity in drug discovery and design. Expert Opin Drug Discov.

[24] AstraZeneca. Experimental in vitro Dmpk and physicochemical data on a set of publicly disclosed compounds (2016) 10.6019/Chembl3301361

[25] Sushko I, Novotarskyi S, Körner R, Pandey AK, Rupp M, Teetz W, Brandmaier S, Abdelaziz A, Prokopenko VV, Tanchuk VY (2011). Online chemical modeling environment (Ochem): web platform for data storage, model development and publishing of chemical information. J Comput Aided Mol Des.

[26] Landrum G. Rdkit: open-source cheminformatics (2006)

[27] Ramsundar B, Eastman P, Walters P, Pande V (2019). Deep Learning for the life sciences: applying deep learning to genomics, microscopy, drug discovery, and more.

[28] Yang K, Swanson K, Jin W, Coley CW, Eiden P, Gao H, Guzman-Perez A, Hopper T, Kelley B, Mathea M (2019). Analyzing learned molecular representations for property prediction. J Chem Inf Model..

[29] Kireev DB (1995). Chemnet: a novel neural network based method for graph/property mapping. J Chem Inf Comput Sci.

[30] Coley CW, Jin W, Rogers L, Jamison TF, Jaakkola TS, Green WH, Barzilay R, Jensen KF (2019). A graph-convolutional neural network model for the prediction of chemical reactivity. Chem Sci.

[31] Kearnes S, McCloskey K, Berndl M, Pande V, Riley P (2016). Molecular graph convolutions: moving beyond fingerprints. J Comput Aided Mol Des.

[32] Duvenaud DK, Maclaurin D, Iparraguirre J, Bombarell R, Hirzel T, Aspuru-Guzik A, Adams RP (2015) Convolutional networks on graphs for learning molecular fingerprints. In Advances in neural information processing systems. pp 2224–2232.

[33] Paszke A, Gross S, Chintala S, Chanan G (2017) Pytorch: tensors and dynamic neural networks in python with strong Gpu acceleration. PyTorch: tensors and dynamic neural networks in python with strong GPU acceleration. 6

[34] Vaswani A, Shazeer N, Parmar N, Uszkoreit J, Jones L, Gomez AN, Kaiser Ł, Polosukhin I (2017) Attention is all you need. In: advances in neural information processing systems. pp 5998–6008.

[35] Bergstra J, Komer B, Eliasmith C, Yamins D, Cox DD (2015). Hyperopt: a python library for model selection and hyperparameter optimization. Comput Sci Discov.

[36] Breiman L (2001). Random forests. Mach Learn.

[37] Polishchuk P (2017). Interpretation of quantitative structure-activity relationship models: past, present, and future. J Chem Inf Model.

[38] Ma J, Sheridan RP, Liaw A, Dahl GE, Svetnik V (2015). Deep neural nets as a method for quantitative structure–activity relationships. J Chem Inf Model.

[39] Oliphant TE (2007). Python for Scientific Computing. Comput Sci Eng.

[40] Pedregosa F, Varoquaux G, Gramfort A, Michel V, Thirion B, Grisel O, Blondel M, Prettenhofer P, Weiss R, Dubourg V (2011). Scikit-learn: machine learning in Python. J Mach Learn Res.

