# Supplementary material for: A self-attention based message passing neural network for predicting molecular lipophilicity and aqueous solubility
Source: J Cheminform. 2020 Feb 21;12:15. doi: 10.1186/s13321-020-0414-z (PMC7035778; doi:10.1186/s13321-020-0414-z)
Supplement: Supplementary file 1 — Additional file 1. The Supporting Information can be found in the supporting documents. The source code and the prepared datasets are available in the SAMPN Github repository (https://github.com/tbwxmu/SAMPN). [file 13321_2020_414_MOESM1_ESM.docx]

**A Self-Attention Based Message Passing Neural Network for Predicting Molecular Lipophilicity and Aqueous Solubility**

*Bowen Tang^1,2^, Skyler T. Kramer ^2^, Meijuan Fang^1^, Yingkun Qiu^1^, Zhen Wu^1,*^ and Dong Xu^2,*^*

^1^Fujian Provincial Key Laboratory of Innovative Drug Target Research, School of Pharmaceutical Sciences, Xiamen University, Xiamen 361000, China

^2^ Department of Electrical Engineering and Computer Science, Informatics Institute, and Christopher S. Bond Life Sciences Center, University of Missouri, Columbia, MO 65211, USA

*Correspondence: [wuzhen@xmu.edu.cn，xudong@missouri.edu](mailto:wuzhen@xmu.edu.cn，xudong@missouri.edu)

**Supporting Information**

| Algorithm 1: Edge-dependent message passing neural network with self-attention |
| --- |

**Table S1.** Algorithm of SANMP model
1: **Input:** clean SMILES in batch

2: **Create molecule graph:** for each molecule mol: list(node_index), list(edge_index), list(n2e),list(e2n) #index mapping n2e: node to edge, e2n: edge to node
3: **Initialize node features:** for each node *a*: F*a* = *f(a)*

4: **Initialize edge features:** for each edge *b*: F*b* = *f(b)*

5: **Initialize message:** message=Re(W_inp_ ∙ concatenate(F*a*, F*b*)) # number equals the edges

6: # Message passing

7: **for** 2~ step - 1:

8: node_neighbors_message = select(message, n2e) #none message-receiving neighbor

9: neighbors_sum= (Node_neighbors_message)

10: message= Re(W_inp_ ∙ concatenate(F*a*, F*b*) + W_h_ ∙ neighbors_sum)

11: #Messsage readout

12: node_neighbors_message = select(message, n2e) #all the neighbors

13: hidden state of nodes = Re(concatenate(F*a*, node_neighbors_message))

14: W_score_, *E_G_* = Self-attention(*G*)
15: Mol_vector = Re (*Wo* ∙ global_pool(G+*EG*))
16: #Prediction
17: F*out* = DenseNetworks(Mol_vector )

| Note: “step” denotes the number of message passing steps; *Wh* denotes the hidden weights; Re denotes the ReLU activate function. |
| --- |

**
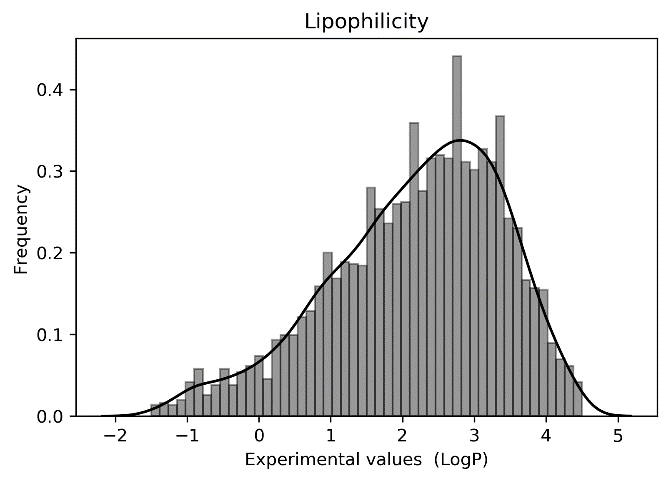

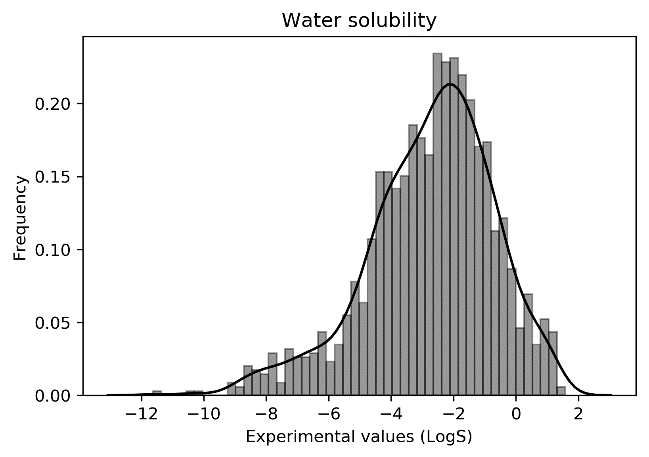
**

B

A

**Fig S1**. Data distributions of lipophilicity and solubility.


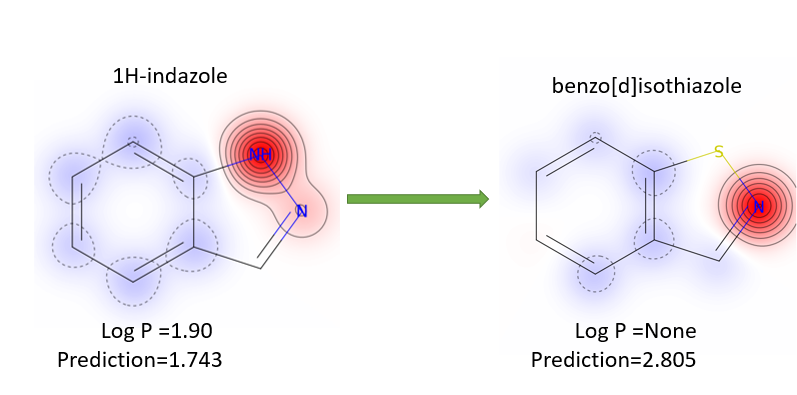


**Fig S2**. Molecular heat map for optimizing the lipophilicity.

**Note S1.** Equations of metrics used to compare performances.

$$MSE={(\sum_{i=1}^{n} \left( Y_{i}-\hat{Y_{i}} \right)^{2})}/n \left( \boldsymbol{1} \right)$$

$$RMSE=\sqrt{MSE} \left( \boldsymbol{2} \right)$$

$$MAE={(\sum_{i=1}^{n} \left| Y_{i}-\hat{Y_{i}} \right|)}/n \left( \boldsymbol{3} \right)$$

$$R^{2}=1-\left( MSE*n \right)/\left( \sum_{i=1}^{n} \left( Y_{i}-\bar{Y} \right)^{2} \right), performed on training set \left( \boldsymbol{4} \right)$$

$$Q^{2}=R^{2}, performed on testing set \left( \boldsymbol{5} \right)$$

$$PC=\frac{n\left( \sum Y_{i}\hat{Y_{i}} \right)-(\sum Y_{i})(\sum\hat{Y_{i}})}{\sqrt{[n\sum{Y_{i}}^{2}-\left( \sum Y_{i} \right)^{2}][n\sum{\hat{Y_{i}}}^{2}-\left( \sum\hat{Y_{i}} \right)^{2}]}} \left( \boldsymbol{6} \right)$$

Note that *Y_i_* refers to the ground truth label, where the hatted variant is the predicted label. Also note that (1), (2), (3), and (6) can be calculated for the training and testing sets and retain the original name of the metric. Conversely, the metrics in (4) and (5) have different names when computed on the training/testing set, respectively.
